# Mechanisms of Hepatocyte Growth Factor Activation in Cancer Tissues

**DOI:** 10.3390/cancers6041890

**Published:** 2014-09-29

**Authors:** Makiko Kawaguchi, Hiroaki Kataoka

**Affiliations:** Section of Oncopathology and Regenerative Biology, Department of Pathology, Faculty of Medicine, University of Miyazaki, 5200 Kihara, Kiyotake, Miyazaki 889-1692, Japan; E-Mail: kawaguchi@med.miyazaki-u.ac.jp

**Keywords:** hepatocyte growth factor, hepatocyte growth factor activator, matriptase, TTSP, HAI-1, HAI-2

## Abstract

Hepatocyte growth factor/scatter factor (HGF/SF) plays critical roles in cancer progression through its specific receptor, MET. HGF/SF is usually synthesized and secreted as an inactive proform (pro-HGF/SF) by stromal cells, such as fibroblasts. Several serine proteases are reported to convert pro-HGF/SF to mature HGF/SF and among these, HGF activator (HGFA) and matriptase are the most potent activators. Increased activities of both proteases have been observed in various cancers. HGFA is synthesized mainly by the liver and secreted as an inactive pro-form. In cancer tissues, pro-HGFA is likely activated by thrombin and/or human kallikrein 1-related peptidase (KLK)-4 and KLK-5. Matriptase is a type II transmembrane serine protease that is expressed by most epithelial cells and is also synthesized as an inactive zymogen. Matriptase activation is likely to be mediated by autoactivation or by other trypsin-like proteases. Recent studies revealed that matriptase autoactivation is promoted by an acidic environment. Given the mildly acidic extracellular environment of solid tumors, matriptase activation may, thus, be accelerated in the tumor microenvironment. HGFA and matriptase activities are regulated by HGFA inhibitor (HAI)-1 (HAI-1) and/or HAI-2 in the pericellular microenvironment. HAIs may have an important role in cancer cell biology by regulating HGF/SF-activating proteases.

## 1. Introduction

The interactions between cancer cells and stromal cells in the tumor microenvironment play a pivotal role in establishing the aggressive characteristics of cancer, such as invasion and metastasis [1]. Indeed, the expression and activity of many growth factors involved in cancer progression are highly dependent on these interactions, with regulation of hepatocyte growth factor (HGF) activity being a typical example of the mutual interaction between cancer cells and stromal cells [2]. HGF, also known as scatter factor (SF), is a multifunctional growth factor that functions as a mitogen and morphogen for a variety of cells through the MET receptor tyrosine kinase, which is encoded by the *Met* proto-oncogene. MET is widely expressed in epithelial cells but is also found in non-epithelial cells, endothelial cells and myoblasts, as well as in hematopoietic system cells and spinal neurons [3,4]. Accordingly, a variety of cancer cells also express MET. To date, a large number of studies demonstrated that HGF/SF-MET signaling is essential for normal development, tissue regeneration and cancer progression [3,4]. HGF/SF is produced by mesenchymal cells and typically activates MET in a paracrine fashion [2,3,4]. The binding of HGF/SF to MET activates the tyrosine kinase activity of MET, which in turn results in the autophosphorylation of tyrosine residues that generates docking sites for proteins that mediate downstream signal transduction [3].

HGF/SF is synthesized as an inactive precursor (pro-HGF/SF) that lacks biological activity, and after secretion is converted to an active mature heterodimer form by proteolytic cleavage [5,6,7,8]. Structurally, HGF/SF is related to other serine proteases such as plasminogen, but lacks protease activities [8,9]. HGF/SF is composed of an α-chain containing the N-terminal domain, four kringle domains, and a β-chain containing a serine protease-like motif. As pro-HGF/SF can bind to MET but does not activate it [9,10], the conversion of pro-HGF/SF to the mature form is critical for establishing HGF/SF-MET signaling. Therefore, detailed knowledge of how this activation step is regulated is important for understanding the pathophysiological roles of HGF/SF and for developing new therapies that target HGF/SF-MET signaling [2,11]. To date, two different activation modalities of pro-HGF/SF have been proposed. One is activation by serum serine proteases, such as HGF activator (HGFA) [11,12,13], while the second involves membrane-anchored serine proteases, such as matriptase [14]. Importantly, these HGF/SF activating proteases are regulated by a cellular serine protease inhibitor, namely HGFA inhibitor (HAI) [13]. This review summarizes the current knowledge regarding proteolytic activation of HGF/SF, its regulation by HAI in cancer tissues, and the possible roles of these proteins in carcinogenesis and cancer progression.

## 2. Enhanced Activation of HGF/SF in Cancer Tissues

HGF/SF is expressed in most tissues throughout the body [12]. In cancer tissues, tumor cells play a role in inducing HGF/SF expression by stromal fibroblasts, whereas fibroblast-derived HGF/SF leads to invasive growth of tumor cells through MET [2,15]. Therefore, HGF/SF typically activates MET in a paracrine fashion, although in some cancers the tumor cells themselves express HGF/SF, which leads to an autocrine loop-type mechanism for MET activation [4]. In preclinical models, autocrine HGF/SF expression correlates with active MET signaling and predicts the efficacy of MET-targeting therapy [16]. As mentioned above, HGF/SF is synthesized as an inactive single-chain pro-HGF/SF. To exert its biological activity, pro-HGF/SF must be converted to the two-chain heterodimeric active form by a single proteolytic cleavage between the Arg^494^ and Val^495^ of the serine protease domain. This step is critical for establishing HGF/SF-induced MET signaling. It should be noted that the molecular form of HGF/SF detected in normal tissues is virtually always the proform [11,17]. However, in contrast to normal tissue, significantly increased levels of the two-chain activated form of HGF/SF are detectable in various cancer tissues [11,18,19,20,21,22], indicating that the HGF/SF activating machinery is up-regulated in cancer tissues.

## 3. Proteases Involved in Pro-HGF/SF Activation

To date, several proteases have been proposed as HGF/SF activators, including HGFA, factor XIIa and XIa, matriptase, hepsin, TMPRSS13, human airway trypsin-like protease (HAT), urokinase-type plasminogen activator (uPA), and tissue-type plasminogen activator (tPA) [11,13,14,23,24,25,26]. These presumed activator proteases can be divided into two groups: serum activators and cellular activators (Table 1). Among serum activators, HGFA shows the most efficient pro-HGF/SF processing activity, which is more than 50-fold more potent than factor XIIa [11,13,14]. Regarding cellular activators, uPA was initially reported to be a major activator of HGF/SF [8,27], although recent studies demonstrated that uPA is not an efficient HGF/SF activator [14,28]. Instead, type II transmembrane serine proteases (TTSPs), such as matriptase, hepsin, TMPRSS13 and HAT, have recently emerged as potential cellular activators of HGF/SF (Table 1). In particular, matriptase shows very efficient pro-HGF/SF processing activity that is comparable or even superior to HGFA [14]. In a recent study by Owen *et al.*, matriptase was shown to be twice as potent as HGFA in the processing of pro-HGF/SF to the mature two-chain form [14]. Hepsin is less active than HGFA, but is still an efficient pro-HGF/SF activator [14,24]. Meanwhile, the efficiency of pro-HGF/SF activation by TMPRSS13 is low and is approximately 90-fold lower than that of HGFA [25] (Table 1).

**Table 1:** HGF/SF-activating proteases.

Proteases	Relative Activity * [Ref.]
Serum proteases	
HGFA	1
Factor XIIa	0.018 [14]~0.02 [11]
Factor XIa	0.011 [11]~0.015 [23]
tPA	<0.00001 [14]
Cellular proteases	
Matriptase	2.07 [14]
Hepsin	0.074 [14]
HAT	0.024 [26]
TMPRSS13	0.012 [25]
uPA	<0.00001 [14]

*: Relative pro-HGF/SF processing activity compared to HGFA.

## 4. Roles for HGF/SF-Activating Proteases in Carcinogenesis and Malignant Progression

HGFA is a factor XIIa-like serine protease that is synthesized and secreted mainly by the liver and circulates in the blood as an inactive proform (pro-HGFA), with concentrations of around 40 nM being detected in human plasma [13]. Presently, there are only two known substrates for HGFA: pro-HGF/SF and its homologous protein, pro-macrophage stimulating protein (pro-MSP; a specific ligand of RON receptor tyrosine kinase) [13,29]. *In vivo*, pro-HGFA activation occurs almost exclusively in response to tissue injury, with thrombin likely mediating the activation through efficient cleavage of the Arg407-Ile408 bond in the presence of negatively charged substances such as dextran sulfate, heparin, and chondroitin sulfate [13]. This situation also likely occurs in cancer tissue, as cancers are “wounds that do not heal” [30], and cancer cells frequently show enhanced pro-coagulant activity [31]. The human kallikrein 1-related peptidases KLK4 and KLK5 are also potent activators of pro-HGFA in the cancer cell microenvironment [32]. The activity of KLK5 towards pro-HGFA is comparable with that of thrombin, while KLK4 activity is only one-fifth that of KLK5 [32]. In addition to circulating HGFA, aberrant HGFA expression has been reported in many types of cancers [13,33,34]. The hypoxic microenvironment found in tumor tissues may also induce enhanced expression of HGFA [22]. More direct evidence for the role of HGFA in cancer tissue was reported in colon cancer, myeloma, and diffuse large B-cell lymphoma, in which a neutralizing antibody against HGFA suppressed HGF/SF activation [18,35,36]. Serum concentrations of activated HGFA were elevated in myeloma patients [37], while increased levels of serum HGFA were also observed in advanced prostatic cancer patients [38]. In breast cancer, tumor tissues from node-positive patients showed higher expression levels of HGFA [39], suggesting that it may be involved in metastatic cancer progression.

Matriptase, also known as membrane-type serine protease 1 (MT-SP1), was initially identified as a novel gelatinolytic protease in conditioned medium from the human breast cancer cell line T-47D, and was subsequently purified from human milk [40,41]. Matriptase is synthesized as an inactive, single-chain zymogen and is widely expressed in various epithelial cells [42,43,44]. Two sequential endoproteolytic cleavages must occur to generate active matriptase, with the first occurring in the amino-terminal SEA domain probably by non-enzymatic hydrolysis after Gly^149^ while the protein is in the Golgi apparatus, and the second within the highly conserved activation cleavage site in the serine protease domain (Arg^614^–Val^615^) at the cell surface [44]. Recent studies demonstrated that matriptase activation is robustly induced by an acidic cellular microenvironment [45,46]. Given that the pH of the extracellular environment of solid tumors is mildly acidic [47], matriptase activation may be accelerated in the tumor microenvironment. Matriptase expression is upregulated in many types of tumor cells including breast, prostate, ovarian, renal, uterine, colon, pancreatic and esophageal, as well as head and neck carcinomas and lymphomas. Furthermore, several studies suggested that overexpression of matriptase in tumor cells correlates with poor patient prognosis [44,48,49,50,51,52,53,54,55,56,57].

Matriptase is also very important in epithelial carcinogenesis. In mice, transgenic matriptase expression in epidermal keratinocytes caused squamous cell carcinoma [58]. Subsequent analysis revealed that deregulated matriptase expression exerted its oncogenic effects through a MET-AKT-mTOR (mammalian target of rapamycin) signaling axis. The activation of this signaling pathway was initiated by matriptase-mediated HGF/SF activation [59]. Indeed, matriptase is uniformly co-expressed in human head and neck squamous cell carcinoma with MET [59]. On the other hand, the roles for matriptase in epithelial carcinogenesis vary considerably depending on tissue type and cellular microenvironment. In intestinal tissue, the absence of matriptase resulted in disruption of epithelial integrity, which eventually promoted invasive adenocarcinoma [60], and this finding is in accordance with the original gene name for matriptase, *ST14* (suppressor of tumorigenesis 14) [61]. The precise mechanism underlying the controversial roles of matriptase in cancer progression remains unknown. In addition to pro-HGF/SF, matriptase has multiple other substrates, such as protease activated receptor 2, pro-prostasin and pro-MSP [48], which may be involved in cellular context- and environment-dependent effects of matriptase on cancer progression.

## 5. HAI Regulation of HGF/SF Activation

Regulation of HGF/SF-activating protease activity in pericellular spaces would be critical for controlling HGF/SF-mediated MET signaling. To date, two cell-associated serine protease inhibitors have been implicated in the pericellular regulation of HGF/SF-activating proteases: HAI type-1 (HAI-1) and HAI-2, which are encoded by the *SPINT1* and *SPINT2* genes, respectively [13]. Both HAI-1 and HAI-2 were initially purified from conditioned medium of the MKN45 human gastric carcinoma cell line as an efficient inhibitor of HGFA [62,63]. These two HAIs are type I transmembrane proteins with two Kunitz-type serine protease domains (bikunin) in the extracellular domain. HAI-2 was also independently purified from placenta tissue and named placental bikunin [64]. The first Kunitz domain is the functional domain in HAI-1 [65], while for HAI-2 two major splicing variants (HAI-2-long and HAI-2-short) have been reported [66]. HAI-2-long contains both Kunitz domains and is the predominant transcript in humans, but in mouse, HAI-2-short, which lacks the first Kunitz domain, is the predominant transcript [66]. Interestingly, although the first Kunitz domain is the functional domain for HGFA inhibition by human HAI-2-long [67], mouse HAI-2-short that carries only the second Kunitz domain was also an efficient HGFA inhibitor [68].

Since the discovery of HAIs, many studies have searched for their target proteases, and it is now clear that HAI-1 and HAI-2 inhibit, not only HGFA, but also other HGF/SF-activating proteases, such as matriptase and hepsin [11,48]. Therefore, the activity of both HAIs would be critical for regulating HGF/SF activation in the pericellular microenvironment.

## 6. Reduced Cell Surface Expression of HAI-1 in Cancer Cells and Its Role in Cancer Progression

HAI-1 is expressed by most epithelial cells, whereas HAI-2 is ubiquitously expressed in normal tissues [69,70]. To date, possible suppressive roles for HAI-1 in cancer progression have been reported. Reduced HAI-1 expression in tumor cells may be involved in the progression of many types of cancers and is associated with a worse prognosis in patients with prostatic, breast, gastrointestinal, ovarian and endometrial cancers [11,21,49,50,53,71,72,73,74,75]. Our recent study showed that membranous HAI-1 immunoreactivity was reduced in the invasion front of oral cavity squamous cell carcinoma (OSCC) [76]. Consequently, HAI-1 knockdown (KD) in the human OSCC cell line SAS resulted in enhanced cellular invasion *in vitro* [76], and a similar result was obtained with the pancreatic adenocarcinoma cell line SUIT-2 [77]. Moreover, a phenotype suggesting epithelial to mesenchymal transition was induced by insufficient HAI-1 expression [73,76,77], which may be mediated by deregulated activity of TTSPs including matriptase [77]. Finally, treatment of HAI-1 KD SUIT-2 cells with recombinant HAI-1 Kunitz domain 1 or engineered overexpression of HAI-1 in a metastatic subline of SUIT-2 abrogated metastatic spreading of these cells *in vivo* [78,79]. Thus, cell surface HAI-1 may be a suppressor of cancer metastasis. While additional studies will be required for a better understanding of the mechanisms by which loss of HAI-1-mediates enhanced metastasis, it is reasonable to speculate that up-regulated HGF/SF-MET signaling may be involved, at least to a certain degree. In fact, HGF/SF and MET were involved in metastatic spreading of SUIT-2 cells [80], and loss of HAI-1 resulted in enhanced pericellular matriptase activity [76,79,81]. Moreover, recombinant HAI-1 could suppress the conversion of pro-HGF/HGF to the mature form in HGF/SF-expressing MRC-5 fibroblasts, while also inhibiting fibroblast-mediated breast cancer cell invasion [82]. On the other hand, paradoxical up-regulation of HAI-1 immunoreactivity also occurs at invasion front of certain cancer tissues [83], which may be caused by hypoxic and oxidative stress [84]. The biological significance of this phenomenon in cancer progression remains unclear. Similar to matriptase, the role for HAI-1 in cancer cell biology may be cellular context- and/or microenvironment-dependent.

HAI-1 may also be a suppressor of epithelial carcinogenesis. In mice, matriptase-mediated skin carcinogenesis was suppressed by co-expression of HAI-1 in keratinocytes [58]. We have reported that in *Apc*^Min/+^ mice, targeted disruption of the Spint1 gene that encodes HAI-1 resulted in significantly increased amounts of tumor formation, whereas activation of HGF/SF in the intestine was enhanced in HAI-1-deficient tumors and non-tumor mucosa [19]. Interestingly, even in mice carrying the wild-type *Spint1* gene, intestinal tumor cells in *Apc*^Min/+^ mice showed significantly decreased HAI-1 immunoreactivity on the cell surface [19].

The molecular mechanisms underlying reduced HAI-1 expression and deregulated activity of TTSPs, particularly matriptase, in tumor cells are complex. Reduced mRNA levels of HAI-1, enhanced ectodomain shedding of HAI-1, or enhanced expression of TTSPs in cells that virtually lack HAI-1 expression could all be possible mechanisms for the HAI-1/TTSP imbalance in tumor cells. In a metastatic subline of SUIT-2, namely S2-CP8, HAI-1 mRNA levels were significantly decreased, while forced re-expression of HAI-1 alleviated the invasion and metastasis of these cells [77,79]. However, the mechanism underlying reduced HAI-1 mRNA expression remains to be determined. In colon cancers, both decreased HAI-1 mRNA levels and enhanced ectodomain shedding likely occur *in vivo*, which could result in significantly decreased amounts of cell surface HAI-1 [83,85]. The enhanced HAI-1 shedding seen in cancer cells is likely mediated by membrane-type 1 matrix metalloprotease (MT1-MMP, MMP14) [19,86] that is known to be involved in the initial tumor formation and invasive growth of cancer cells [87]. Meanwhile, in B cell lymphomas that barely express HAI-1, overexpression of matriptase can occur, which results in enhanced shedding of active matriptase due to insufficient amounts of HAI-1 [57]. In any case, insufficient HAI-1 relative to TTSPs likely results in deregulated pericellular activities of TTSPs, which may alter the biology of cancer cells significantly.

## 7. Epigenetic Silencing of HAI-2 in Cancer Cells and Its Role in Cancer Progression

Like HAI-1, HAI-2 downregulation and its correlation with disease progression have been observed in many cancers, including malignant brain tumors [88,89], renal cell carcinoma [21,90,91,92], hepatocellular carcinoma [93], gastric adenocarcinoma [94], esophageal squamous cell carcinoma [95], ovarian carcinoma [72], prostate adenocarcinoma [96], and breast carcinoma [39]. It should be noted that the major molecular mechanism underlying HAI-2 downregulation in cancer cells appears to be hypermethylation in the promoter region of the *SPINT2* gene [89,91,92,93,95]. In addition, a HAI-2 missense substitution (P111S) was reported in one renal cell carcinoma cell line, and while restoration of wild-type HAI-2 expression in this cell line reduced *in vitro* colony formation, the P111S mutant had no significant effect [91]. Therefore, the HAI-2 (*SPINT2*) gene is a candidate for a novel tumor suppressor, although further studies will be required to determine the regulatory roles of HAI-2 in pericellular activation of HGF/SF and induction of MET signaling, as well as to describe the effect of HAI-2 loss on MET signaling in cancer cells.

## 8. Conclusions and Future Perspectives

**Figure 1:**
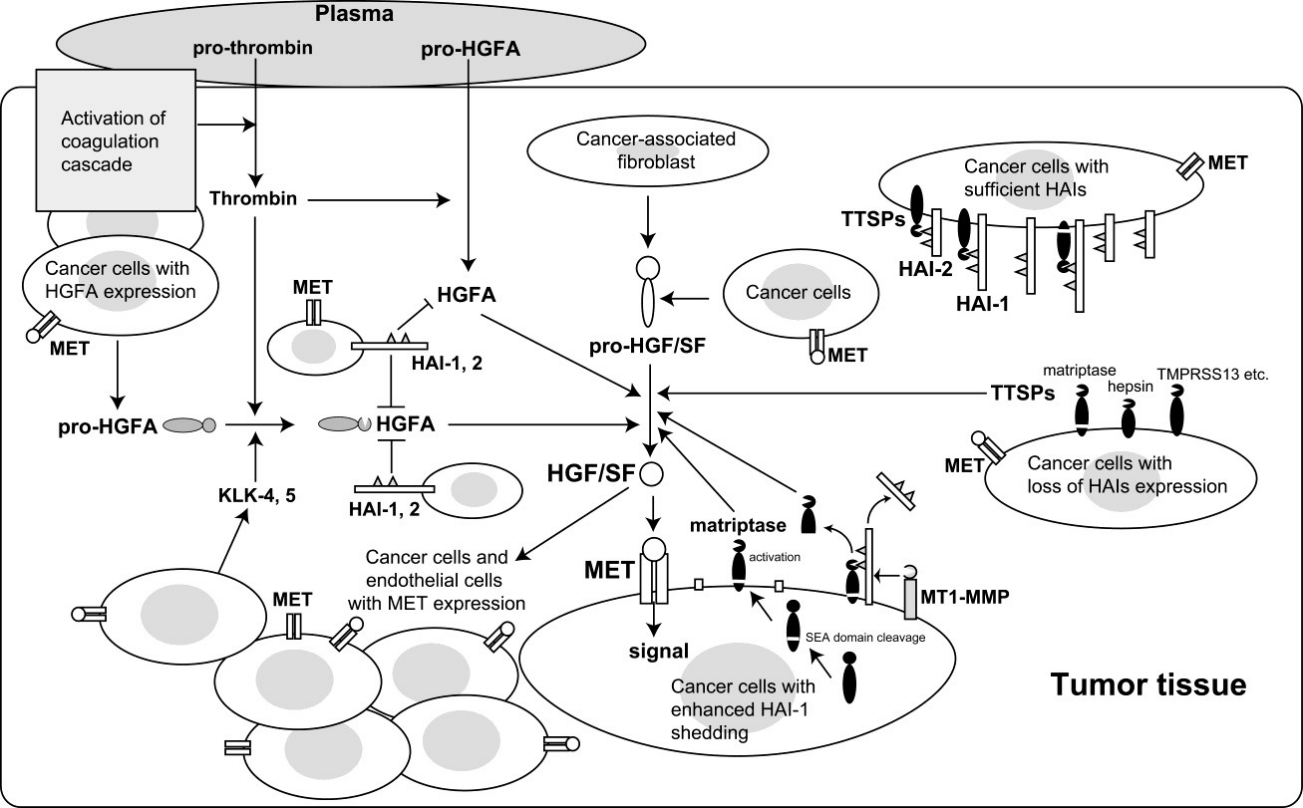
Hypothetical model of HGF/SF activation in cancer. There are two major pathways in the activation of HGF/SF in tumor tissue. One is HGFA-mediated pathway that is closely linked to activation of the coagulation cascade. Another pathway is TTSPs-mediated one, in which matriptase likely has a signiificant role. Cellular Kunitz-type serine protease inhibitors, HAI-1 and HAI-2, have crucial roles in the regulation of these HGF/SF-activating proteases in tumor tissues.

Considering the important roles of MET signaling in cancers, more attention should be paid to the mechanisms of processing and activation of its specific ligand, HGF/SF, in the pericellular microenvironment of tumor tissues. In this review we summarized evidence regarding the roles of HGF/SF-activating proteases and the critical HGF/SF regulators, HAI-1 and HAI-2, in carcinogenesis and cancer cell biology. Our hypothetical model for the molecular interactions and cascades that occur during HGF/SF activation in cancer tissues is shown in Figure 1.

As the HGF/SF-activating proteases discussed in this review are likely to be involved in processing other growth factors, such as MSP [13], platelet-derived growth factor (PDGF)-C and PDGF-D [97,98], the pericellular HGF/SF-activating machinery may be a promising molecular target for innovative cancer therapies. Indeed, several basic and preclinical attempts to target HGF/SF-activating proteases by recombinant HAI-1 Kunitz domain or synthetic inhibitors have been reported [78,99,100,101]. Thus, there is clearly a need for further studies for a better understanding of the roles of HGF/SF-activating proteases and their inhibitors in carcinogenesis and cancer progression.
